# Combined Checkpoint Inhibition and Chemotherapy: New Era of 1^st^-Line Treatment for Non-Small-Cell Lung Cancer

**DOI:** 10.1016/j.omto.2019.02.001

**Published:** 2019-03-19

**Authors:** Chongkai Wang, Prakash Kulkarni, Ravi Salgia

**Affiliations:** 1 Department of Medical Oncology and Therapeutics Research, City of Hope National Medical Center, Duarte 91010, CA, USA

**Keywords:** non-small cell lung cancer, PD-1, PD-L1, checkpoint blockade, immunotherapy, chemotherapy, combination therapy

## Abstract

Platinum-based chemotherapy has long been the first-line treatment of choice for metastatic non-small-cell lung cancer (NSCLC) patients who lack targetable gene mutations. The arrival of checkpoint blockade has led to a vast shift in the treatment landscape of NSCLC. Among NSCLC patients with PD-L1 expression in ≥50% of tumor cells, treatment with pembrolizumab leads to a superior progression-free and overall survival compared to platinum-doublet chemotherapy in the first-line setting. Furthermore, the addition of pembrolizumab to standard chemotherapy of pemetrexed and a platinum-based drug resulted in significant longer progression-free survival and overall survival irrespective to PD-L1 expression. In this review, we focus on the molecular rationale for the combination therapy and the results of completed clinical studies.

## Main Text

Lung cancer remains one of the leading causes of deaths from cancer worldwide, with ∼80% of all cases presenting as non-small-cell lung cancer (NSCLC).^1^, ^2^ Currently, the standard first-line treatment for patients with advanced NSCLC without targetable driver mutations is platinum-based, two drug chemotherapy regimens, which achieved a median overall survival of 10.3 months. The addition of bevacizumab to chemotherapy showed modest benefit, with an improved overall survival of 12.3 months.^3^ Despite extensive investigations, the addition of a third cytotoxic agent to platinum-doublet regimen failed to demonstrate improvement in progression-free survival (PFS) or overall survival (OS) over platinum-doublet chemotherapy alone in various clinical trials.^4^

A phase I study of pembrolizumab in patients with advanced NSCLC has shown robust efficacy, including a median OS of 22.3 months in treatment-naive patients and a median OS of 34.9 months in patients whose PD-L1 tumor proportion score (TPS) is ≥50%.^5^, ^6^ This superior efficacy was confirmed by a phase 2 and 3 study, where treatment with pembrolizumab prolonged OS by 2–4 months in PD-L1-positive (TPS ≥ 1%) NSCLC patients who progressed after platinum-based chemotherapy versus standard-of-care treatment.^7^

There is now increasing evidence that suggests that the anti-tumor effects of chemotherapy is mediated not only through cytotoxic effects but also through modulating tumor immunity, including inducing immunogenic cell death, enhancing tumor antigen presentation, as well as inhibiting regulatory T cells, and the elimination of myeloid-deprived suppressor cells (MDSCs).^8^, ^9^, ^10^, ^11^ Preclinical studies have demonstrated that chemotherapies can stimulate the immune system, causing anti-tumor immunity that contributes to the efficacy of these drugs.^12^, ^13^ In addition, early clinical trials for combining PD-1 or PD-L1 blockade with chemotherapy showed superior anti-tumor activity in first-line setting for advanced NSCLCs.^14^, ^15^, ^16^ These results lend credence to the argument that combination of chemotherapy and immunotherapies is the next step for cancer treatment.

### Immunotherapy for Non-Small-Cell Lung Cancer

Platinum-based chemotherapy has long been the first-line therapy for most patients with metastatic NSCLC without a targetable driver mutation, with a median OS of approximately 12 months.^17^ Approximately one-half of patients with advanced NSCLC never receive second-line therapy due to rapid disease deterioration during progression.^18^ The trial from KEYNOTE-001 demonstrated pembrolizumab was associated with a median PFS of 12.5 months and a median OS of 22.1 months for treatment-naive patients and 10.6 months for previously treated patients (PD-L1 TPS ≥ 1%).^19^ Compared with docetaxel, treatment with pembrolizumab was associated with prolonged OS in previously treated, PD-L1 TPS ≥ 1%, advanced NSCLC.^7^ These data confirmed pembrolizumab provides superior OS benefit for PD-L1-positive untreated and previously treated NSCLC. When administrated as first-line therapy, pembrolizumab provided significantly prolonged PFS and OS over chemotherapy for metastatic NSCLC with PD-L1 TPS ≥ 50%.^20^, ^21^ Similarly, in the KEYNOTE-042 trial, patients with PD-L1 TPS ≥ 1%, the superiority of pembrolizumab over platinum-based chemotherapy is confirmed in treatment-naive metastatic NSCLC without epidermal growth factor receptor (EGFR) or anaplastic lymphoma kinase (ALK) driver mutations.^22^ These data provide strong rationale to extend pembrolizumab monotherapy as a standard first-line treatment for PD-L1-positive metastatic NSCLC.

### Immunomodulation by Platinum-Based Chemotherapy

Chemotherapy stimulates anti-tumor immunity in two major ways: inducing immunogenic cell death (ICD)^1^ and disrupting the immune-suppressive tumor microenvironment that tumor uses to escape immune surveillance.^2^ On cellular lever, the hallmark of ICD includes calreticulin translocation to cell membrane, which serves as a signal to dendritic cells (DCs) to clear the dying cell, and the release of ATP and high mobility group box protein 1 (HMGB1), which stimulate DC activation and maturation.^23^ Treatment with oxaliplatin stimulates calreticulin exposure and HMGB1 release in colon cancer cells, and injection of oxaliplatin-treated cells into mice induced an anti-tumor immune response, which is mediated through HMGB1-TLR4 (Toll-like receptor) axis.^24^ Similarly, oxaliplatin-mafosfamide combination stimulated calreticulin exposure and HMGB1 release in lung adenocarcinoma models.

**Figure 1:**
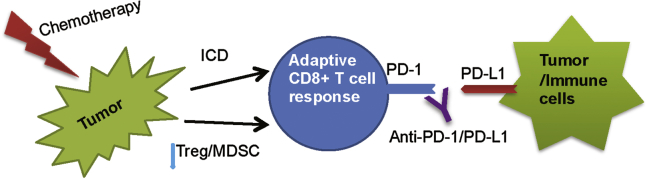
Chemotherapy Modulates Anti-tumor Immune Response and Synergy with Checkpoint Blockade Various chemotherapy drugs can promote anti-tumor immune response by two major ways: (1) induction of immunogenic cell death, resulting in the evolution of tumor-specific CD8+ T cells, and (2) downregulation of suppressive immune cells, such as Tregs and MDSCs in the tumor microenvironment. Combination with checkpoint blockade prevents cytotoxic T cell exhaustion and leads to durable immune-mediated anti-tumor immune response.

Platinum-based chemotherapy modulates the composition and activity of tumor-infiltrating immune cells, which include increased infiltration of CD8+ T cells, maturation of antigen-presenting cells (APC), and downregulation of regulatory T cells (Tregs) and MDSCs at the tumor sites.^25^, ^26^, ^27^ Increased CD8+ T cell infiltration and CD8+ T cell:Treg ratio was also observed in oxaliplatin-mafosfamide-treated mice, which sensitize the tumor to immune checkpoint blockade.^28^ In addition, exposure to platinum chemotherapeutic drugs inhibit expression of programmed death-receptor-ligand 2 (PD-L2) on both tumor cells and dendritic cells (DCs), resulting in enhanced T cell stimulation and antigen reorganization.^29^ Other immune-stimulating effects of platinum-based drugs include increased major histocompatibility complex (MHC) class I expression and sensitizing tumor cells to granzyme-B-mediated cytotoxic T cell killing.^12^, ^25^ Based on these findings, it is well acknowledged that platinum-based chemotherapy promotes the development of tumor-specific immunity and combining with immune checkpoint blockade may boost the function of effector T cells (Figure 1).

### Synergy between Immunotherapy and Platinum-Based Chemotherapy

Preclinical studies have confirmed that platinum-based chemotherapy elicits tumor CD8+ T cell infiltration and sensitized tumor to immune checkpoint blockade, which resulted in a durable control of cancer.^28^ More importantly, clinical studies have showed promising immunogenic effects of platinum. Compared to chemotherapy alone, combination therapy with ipilimumab and carboplatin and paclitaxel resulted in an improved PFS in a randomized phase II study in NSCLC in first-line setting.^30^ A similar efficacy was also observed in a phase II trial of small-cell lung cancer.^31^ A randomized, controlled phase II trial has showed that addition of pembrolizumab to carboplatin and pemetrexed improved efficacy and resulted in a superior benefit-to-risk profile in chemotherapy-naive, metastatic NSCLC patients irrespective of PD-L1 expression, with a median PFS in the pembrolizumab combination group of 24 months compared to 9.3 months in the chemo alone group.^32^, ^33^ These findings were validated by the phase III KEYNOTE-189 trial in first-line setting: the triplet combination regimen significantly prolonged PFS and OS compared with cisplatin or carboplatin plus pemetrexed chemotherapy alone in metastatic nonsquamous NSCLC patients without sensitizing EGFR or ALK mutations.^34^ The superior efficacy of this triplet regimen was also observed in a phase III trial of untreated metastatic, squamous NSCLC.^35^ Largely based on those trials, the Food and Drug Administration (FDA) approved the use of pembrolizumab along with pemetrexed and carboplatin as a first line of treatment for patients with metastatic, nonsquamous NSCLC in September 2017. More interestingly, the IMpower150 trial demonstrated that adding PD-L1 inhibitor atezolizumab to bevacizumab plus chemotherapy with carboplatin and paclitaxel in first line setting significantly improved PFS and OS among patients with advanced nonsquamous NSCLC, regardless of EGFR or ALK mutations and PD-L1 expression status.^36^ This suggests that blocking immunosuppression mediated by vascular endothelial growth factor may enhance the efficacy of immunotherapy.

Several phase I and II clinical trials of chemotherapy combining with checkpoint blockade enrolling patients with different stages and histologies are currently ongoing (Table 1). These trials are investigating (1) whether combining checkpoint blockade with new chemotherapy drugs can help, (2) whether combining checkpoint blockade with standard chemotherapy is superior in adjuvant and neoadjuvant setting, (3) whether combining checkpoint blockade with standard chemotherapy is effective in earlier stages of disease, and (4) whether combining checkpoint blockade with other chemotherapy or targeting therapy drugs will overcome resistance to checkpoint inhibitor treatment. Their primary end points include safety and toxicity, maximum tolerated dose, objective response rate (ORR), PFS, OS, and alterations of immune response. Results from these trials will better establish the efficacy and toxicity of this regimen to better understand the immune evolution following combination treatment, to explore regimens that can overcome checkpoint inhibition resistance, and may extend this approach to earlier stages.

**Table 1:** Ongoing Studies of Immunotherapy in Combination with Chemotherapy or Targeting Therapy in Lung Cancer

NCT Number	Study Phase	Disease Stage	Trial Design	Line of Therapy
NCT03233724	phase I and II	inoperable stage I–IV NSCLC (n = 75)	tetrahydrouridine-decitabine (THU-DAC) with pembrolizumab	1^st^ line
NCT03823625	phase II	stage IIIB not amenable to radical treatment or stage IV or recurrent squamous-cell lung carcinoma (SqLC) (n = 112)	nivolumab plus ipilimumab versus platinum-based chemotherapy plus nivolumab	1^st^ line
NCT03801304	phase II	stage IV NSCLC	atezolizumab plus vinorelbine	2^nd^ line
NCT03568097	phase II	stage IV SCLC (n = 55)	avelumab + standard 1^st^ line chemotherapy (cisplatin or carboplatin + etoposide)	1^st^ line
NCT02581943 (immune markers)	phase II	recurrent or stage IIIB–IV NSCLC (n = 40)	pembrolizumab versus pembrolizumab + carboplatin + paclitaxel	2^nd^ and 3^rd^ line
NCT03257722	phase Ib and II	NSCLC patients who have failed immune checkpoint inhibitor (n = 40)	pembrolizumab + idelalisib	any line
NCT03689855	phase II	stage IV NSCLC patients progressed on any immune checkpoint blocker (n = 21)	ramucirumab + atezolizumab	any line
NCT02638090	phase I and II	stage IV NSCLC (n = 100)	pembrolizumab + vorinostat	any line

### Predictive Biomarkers of Response to Immunotherapy in NSCLC

Various tissue- and plasma-based tests have been investigated with the aim of identifying biomarkers related to benefit from checkpoint blockade. A recent study using whole-exome sequencing demonstrated higher tumor mutation burden (TMB) was associated with improved objective response rate and prolonged PFS in NSCLC patients treated with pembrolizumab.^37^ This observation was confirmed by Foundation One database analysis, where high TMB (>15 mutations/MB) was associated with longer clinical response in NSCLC patients treated with PD-1 or PD-L1 inhibitors.^38^ In addition, POPLAR trial and Checkmate 026 trial showed similar observation, where higher TMB was associated with improved efficacy in NSCLC patients treated with PD-1 or PD-L1 inhibitor in the first- and second-line setting.^39^, ^40^

Historically, PD-L1 expression has been used as a cutoff for selecting patients in numerous clinical trials. Although studies have demonstrated that high PD-L1 expression increases the likelihood of response to PD-1 blockade therapy, responses in tumors lacking PD-L1 expression was also reported. Interestingly, the heterogeneity of PD-L1 expression in tumor microenvironment (TME) may be a key factor affecting the efficacy of immunotherapy. PD-L1 expression on tumor-infiltrating immune cells (macrophages and dendritic cells), but not tumor cells, correlated with responses,^41^ which may explain the lack of efficacy of checkpoint blockade in a subset of PD-L1-high patients.

High tumor-infiltrating lymphocyte (TIL) presence has been associated with superior prognosis in multiple solid tumors, including NSCLC.^42^ In a retrospective study of NSCLC patients treated with nivolumab, high CD8^+^PD-1^low^ TILs in pre-treated samples was associated with prolonged PFS.^43^ Interestingly, in a phase II trial of nivolumab for high-risk stage IB–IIIA NSCLC patients in neoadjuvant setting, comparing to pre-treatment, an influx of PD-1+CD8+ T cell infiltration in TME after treatment was associated with clinical response.^44^ In a phase II trial of atezolizumab versus docetaxel for patients with previously treated NSCLC, increased PD-L1 expression on tumor cells and tumor-infiltrating immune cells and high T-effector (Teff)-associated gene expression was associated with improvement in OS.^45^ The Teff gene expression signature was further confirmed in a subsequent phase III trial, which showed high Teff expression identified more patients who are likely to experience a clinical benefit with atezolizumab than using PD-L1 expression as a biomarker.^46^

In sum, multiple studies have suggested TMB as a strong biomarker for clinical benefit from PD-1 or PD-L1 inhibitors. The heterogeneity of PD-L1 expression and dynamic changes of CD8+ TILs and its interplay with other components of TME (such as Teff) during disease course needs more thorough study to establish a clinical cutoff for its prognostic and predictive value. Meanwhile, other assays that have demonstrated predictability of efficacy of immunotherapy in melanoma and other solid tumors, such as T cell clonality, immunoscore, immunophenoscore, and others, are under investigation in NSCLC.

### Resistance to Immunotherapy in NSCLC

Despite the unparalleled durable response with checkpoint inhibitors, the majority of patients do not benefit (primary resistance) and some patients relapse after an initial response (acquired resistance). To this point, most studies of mechanisms of resistance to immunotherapy were performed in melanoma, which includes tumor cell intrinsic and extrinsic factors. The tumor-cell-intrinsic factors include low neoantigen, deficient antigen presentation, insensitivity to interferon gamma pathway signaling, as well as genetic T cell exclusion, such as overactivation of wnt and β-catenin signaling. Tumor-cell-extrinsic factors include upregulation of alternative inhibitory checkpoint molecules; infiltration of other immunosuppressive cell populations, such as macrophages and regulatory T cells; as well as lack of specific anti-tumor T cell responses.^47^ In NSCLC, trials have shown that patients with EGFR mutant tumors do not benefit from PD-1 or PD-L1 inhibitors.^48^, ^49^ Further studies have demonstrated that EGFR mutant and ALK-rearrangement-positive NSCLC tumors lacked concurrent CD8+ T cell infiltration and PD-L1 expression, which is associated with inferior efficacy of PD-1 or PD-L1 inhibitors in this subset of the population.^50^ Quite contrary to this observation, a recent phase III trial showed significant efficacy in this subset of patients treated with atezolizumab and bevacizumab plus chemotherapy with carboplatin and paclitaxel.^36^ Another phenomenon worth noting is hyperprogressive (HP) disease in patients treated with immunotherapy, which occurs in about 17% metastatic NSCLC patients after starting checkpoint inhibitors.^51^ One possible explanation is accumulation of M2-like macrophages in pre-treatment tumors, and the interaction between Fc-Fcγ receptor binding of Nivolumab and macrophages facilitated tumor progression.^52^ Further investigations are warranted to interrogate the mechanisms underlying resistance and hyperprogressive disease of checkpoint inhibitors.

### Conclusions and Future Perspectives

Platinum-based chemotherapy has been the treatment of choice of advanced NSCLC for years in the first-line setting. The development of checkpoint inhibitors targeting PD-1 or PD-L1 pathway has drastically changed the treatment paradigm of metastatic NSCLC. The extensive clinical investigations of combination of pembrolizumab with carboplatin and pemetrexed have facilitated the FDA approval of the first combination of chemotherapy and immunotherapy for patients with lung cancer. Despite having more therapeutic opportunities, this has also increased the complexity of therapeutic selection. In the age of precise medicine, the most urgent challenge that remains is the lack of biomarkers to predict which patient may benefit the most from combination of chemotherapy and checkpoint blockade and which patient may experience hyperprogressive tumor progression. Growing evidence suggests that the genomic context of the tumor may have a critical influence on the tumor microenvironment, thus impacting the efficacy of immunotherapy. This can be partially interrogated via next-generation sequencing where the tumor genetics and efficacy of selected treatment can be studied. In addition, the dissection of immune infiltration of TME via high-resolution multiplex technique, such as CyTOF, can provide a more fundamental understanding of the immune component of TME and its association with treatment selection and efficacy. Another increasing concern is the acquired resistance to therapy after initial tumor regression. Additional investigations on elucidating the mechanisms underlying acquired mechanism(s) that are currently underway may help in developing combination strategies that benefit patient in the long term.

## Author Contributions

C.W. and P.K. contributed literature search and review, writing, graphical design, and editing; R.S. contributed conception and design and editing. All authors read and approved the final manuscript.

## Conflicts of Interest

The authors declare that there is no conflict of interest.
